# Pathways to care for patients with schizophrenia and severe social impairment

**DOI:** 10.1192/j.eurpsy.2025.772

**Published:** 2025-08-26

**Authors:** I.-M. Mølstrøm

**Affiliations:** Psychiatry East, Region Sealand Psychiatry, Research Unit for Early Detection and Psychopathology, Roskilde, Denmark

## Abstract

**Introduction:**

This presentation sheds light on a group of patients with schizophrenia that are often overlooked. These minority groups include homeless patients and patients with very poor social functioning. Homeless patients with schizophrenia often have complex problems and are difficult to diagnose and treat. While many resources are allocated to early detection and prevention of psychosis programs, few resources are spent on treating the patients who have fallen through the cracks of society and various support systems. These patients are undeniably among the most severely ill psychiatric patients of our time. Patients who are not homeless (domiciled patients) with schizophrenia and very poor social functioning are also often challenging to treat, and they often end up living an isolated existence in their own homes.

**Objectives:**

This presentation will examine the pathways and barriers to care for patients with schizophrenia who are homeless or have serious social function impairment.

**Methods:**

The presentation is based on results from an explorative, cross-sectional study of 85 patients with schizophrenia spectrum diagnosis and severely impaired social function, who either were homeless or domiciled but in need of an outreach team to secure continuous treatment. The study was conducted in Copenhagen in 2020-2024.

**Results:**

We found striking delays in both groups, but most severe in the homeless group. We found a duration of untreated psychosis of ten years and a service delay (period from first contact to psychiatry until a schizophrenia spectrum diagnosis) of four years. Possible reasons for the alarming delays will be discussed, including diagnostic overshadowing and the difficult-to-recognize presentations of schizophrenia. Potential solutions for moving forward will also be highlighted, such as rekindling the diagnostic process.

**Conclusions:**

The overall results from this presentation indicate that there are highly vulnerable minority groups of patients with schizophrenia who have not benefited from the improvements in diagnosis and treatment that we have witnessed in psychiatry in the last 100 years. Acquiring a better understanding of these patients’ conditions, symptom presentations, and barriers to timely diagnosis is highly warranted.

**Disclosure of Interest:**

None Declared

